# The impact of premenstrual disorders on work disruptions among working women: A cross-sectional study

**DOI:** 10.18502/ijrm.v22i2.15712

**Published:** 2024-03-25

**Authors:** Ziba Loukzadeh, Nazila Eslamy, Marziyeh Dehghan, Amir Houshang Mehrparvar

**Affiliations:** Industrial Diseases Research Center, Center of Excellence for Occupational Medicine, Shahid Sadoughi University of Medical Sciences, Yazd, Iran.

**Keywords:** Premenstrual syndrome, Premenstrual dysphoric disorder, Work performance, Working women, Productivity.

## Abstract

**Background:**

Physical and emotional manifestations of premenstrual disorder cause increased absenteeism, decreased productivity, and decreased work-related quality of life.

**Objective:**

Due to the relatively high prevalence of premenstrual disorders in Iran and limited studies on its work-related problems, this study investigated the relationship between premenstrual disorders and work performance in working women.

**Materials and Methods:**

This cross-sectional study was conducted on 358 working women (teachers and industrial workers) in Yazd, Iran, from July 2019 to January 2020. Data were collected using premenstrual symptom screening tool, the work productivity and activity impairment (a modified version), and functional work capacity questionnaires. Women were classified into 2 groups: women with and without premenstrual disorders. Productivity, functional capacity, and ability to perform activities of daily living were compared between groups.

**Results:**

Among 358 participants, 121 women (33.8%) had premenstrual disorders. The prevalence of premenstrual disorders was significantly higher in teachers than workers (0.41% vs. 24.7%, respectively) (p = 0.002). The work results showed a worse score in the group with premenstrual disorder than the other group and teachers compared to workers (p < 0.001).

**Conclusion:**

This study showed a significant association between premenstrual disorders and worse work productivity, functional work capacity, and ability to perform activities of daily living. Teachers had a higher prevalence of premenstrual disorders and worse work performance than workers, which can be due to higher education levels, work stress, more complex tasks, and increased work responsibility in teachers.

## 1. Introduction

90% of women have experienced at least one or more signs or symptoms of Premenstrual syndrome (PMS) (1). A meta-analysis in Iran reported that the overall prevalence of PMS in Iran was 70.8%; hence, a significant proportion of women may face premenstrual problems every month (2).

PMS causes many costs: direct costs include medical care (i.e., diagnosis and treatment) and indirect costs due to absenteeism, loss of income, less productivity, and decreased work-related quality of life. On the other hand, these costs may be underestimated due to the taboo nature of menstruation, that is, women may be ashamed to disclose their premenstrual problems. Severe indirect costs include reduced quality of life due to physical and psychological complications and its effects on interpersonal relationships (3). A study was conducted on 32,748 women, self-reporting about the loss of productivity related to menstruation, 13.8% of women reported absenteeism during the menses, and 3.4% were absent almost every month. The mean absenteeism was reported 1.3 days per year. 80.7% of participants reported presenteeism (the loss of productivity while present at work) and decreased productivity equal a mean of 23.2 days of total lost productivity per year. Among those who announced their absence to the employer, only 20.1% mentioned menstrual problems as the cause of sickness (4). It has been seen that PMS may affect sleep quality and 1/3 ${\;}^{\text{rd}}$ of reproductive age women complain of sleep disorders related to the menstrual cycle (5).

Although the physical and emotional effects of PMS/premenstrual dysphoric disorder (PMDD) have been shown in previous research, studies in this area are limited and inconsistent. Even in some studies, no relationship between PMS/PMDD and work situations has been reported (1).

Therefore, due to the relatively high prevalence of PMS in Iran, its associated costs, increasing number of female workers, and very few studies on the effects of premenstrual disorder in the workplace, this study aimed to assess the association between premenstrual disorders and work disruption in working women.

## 2. Materials and Methods

In this cross-sectional study, 404 participants were enrolled among working women in the morning shift of Almas Sabz Pistachio Sorting plant factory and teachers in Yazd, Iran, from July 2019 to January 2020. The selection of participants was done using census sampling for the factory workers and cluster sampling for teachers. Women with endometriosis, uterine tumors, depression, anxiety, inflammatory bowel diseases, pregnancy, breastfeeding, or postmenopausal status were not included. Additionally, women who had given birth or had an abortion within the past year were also excluded.

For all the participants, 4 questionnaires were completed by a trained female doctor asking women as following:

1) The first questionnaire consisted of questions about demographic/lifestyle/menstruation characteristics such as age, work experience, marital status (married or single/divorced/ widowed), number of children, level of education (primary or secondary/ university), exercise (yes, no/sometimes), age of onset of menstruation (menarche), the regularity of the cycle, the menstrual bleeding days, and the length of the entire menstrual cycle.

The 2 ${\;}^{\text{nd}}$ questionnaire was the Persian version of the standard premenstrual symptom screening tool (2). Premenstrual symptom screening tool consists of 19 questions in 2 parts; the first 14 questions evaluates mood, physical and behavioral symptoms, and the effect of these symptoms on the lives of people was assessed by the last 5 ones.

2) The answers to each question have 4 options (not at all, mild, moderate, and severe) and are scored from 0–3.

The following 3 conditions must be met to diagnose moderate or severe PMS:

i) From questions 1–4, at least 1 answer should be moderate or severe.

ii) Besides to the first criterion, from questions 1–14, at least 4 answers should be moderate or severe.

iii) There should be one moderate or severe answer in a section of the symptom effects on life (last 5 questions).

The following 3 conditions must be met to diagnose PMDD:

i) From questions 1–4, at least one answer should be severe.

ii) Besides the first criterion, from questions 1–14, at least 4 answers should be moderate or severe.

iii) There should be at least 1 severe answer in the section on the effect of symptoms on life.

3) A modified version of the work productivity and activity impairment questionnaire was used to assess menstrual-related issues (6). We used the last 2 questions of this questionnaire, a visual score from 0–10; the participants presented their views on the effect of PMS problems on work productivity and ability to perform routine activities of daily living (ADL). Choosing a higher score indicates the worse impact of PMS on work productivity and the ability to perform ADL.

4) The 4 ${\;}^{\text{th}}$ questionnaire was the functional work capacity questionnaire consisting 8 questions and has been used to evaluate the functional defects caused by PMDD and PMS (3). These 8 questions are about judgment, reduced efficiency, difficulty completing daily job tasks, forgetfulness, more accidents, easily distracted, negative and hostile feelings toward others, and clumsiness. Moreover, the answers were yes/no, and the final score was from 0–8. A higher score indicates more work capacity.

The prevalence of PMS symptoms in the mild PMS group was higher compared to women without PMS, but this difference was not statistically significant (p = 0.24), so they were classified as non-PMS group.

After identifying women with PMS/PMDD as a PMS group (n = 121) and other women (No PMS or with mild PMS) as a non-PMS group (n = 237), demographic data, the effect of disorders on ADL, and productivity were compared between PMS and non-PMS groups.

### Ethical considerations

The study protocol was approved by the Ethics Committee of Shahid Sadoughi University of Medical Sciences, Yazd, Iran (Code: IR.SSU.MEDICINE.REC.1398.087). Written informed consent obtained from all participants and they were assured that their information would be kept confidential.

### Statistical analysis

Statistical Package for the Social Sciences, version 16.0, SPSS Inc., Chicago, Illinois, USA (SPSS) was used. Student *t* test and Mann-Whitney test were used to compare parametric and nonparametric quantitative variables between non-PMS and PMS groups; the Chi-square and Fisher's Exact tests were used to compare qualitative variables between non PMS and PMS groups. Using linear regression, we assessed the relationship between PMS status and individual and work-related factors (functional work capacity, ADL, and work productivity). ANOVA test was used to compare quantitative variables between the 3 groups of non PMS, PMS, and PMDD. A p-value of less than 0.05 was considered significant.

## 3. Results

Initially, 404 women were eligible to enter the study. Out of them, 46 women who had issues such as endometriosis, uterine tumors, depression, anxiety, inflammatory bowel diseases, pregnancy, breastfeeding, postmenopausal status, and given birth or an abortion within the past year were excluded from the study.

Finally, 358 women (158 plant workers from the pistachio sorting plant and 200 teachers) were included in this study. The participants in the study ranged in age from 18–51 yr. Out of them, 237 women (66.2%) were classified as “non-PMS group”, while 121 (33.8%) were classified as “PMS group”. Furthermore, the prevalence of PMS was significantly higher among teachers (41.0%) compared to sorting workers (24.7%) (p = 0.002).

No significant differences were observed between the 2 groups in terms of age, work experience, body mass index (BMI), number of children, menarche age, length of the menstrual cycle, status of the menstruation cycle, duration of mensuration, marital status, oral contraceptive pill, and antidepressant use. The prevalence of irregular exercise/lack of exercise in the PMS group was significantly higher than in the non-PMS group (p = 0.01). In terms of education level, these 2 groups had a significant difference (p = 0.01) (Table I).

Results showed that 41.0% of teachers and 24.7% of workers complained about PMS/PMDD, and the difference was statistically significant (p = 0.002).

According to our research, there were significant differences between the 2 groups in terms of work productivity, ADL, and functional work capacity (p < 0.001) as shown in table II. Specifically, when comparing work productivity, ADL performance, and functional work capacity, teachers consistently achieved worse scores compared to workers in all 3 areas (p < 0.001).

After adjusting for confounding variables, the regression analysis revealed a statistically significant relationship between PMS status and work productivity, ability to perform ADL, and functional work capacity (p < 0.001). Furthermore, when comparing teachers and workers, teachers exhibited lower scores in all 3 indicators (p < 0.001) (Table III).

To investigate the effect of the severity of premenstrual symptoms on the work outcomes, the participants in 3 groups, PMMD, PMS, and non-PMS, were compared. It was observed that the PMDD had the worst negative impact on functional work capacity, the ability to perform daily activities, and work productivity (PMDD [23, 6.4%], PMS [98, 27.4%], and non-PMS [237, 66.2%], p < 0.001).

**Table 1:** Demographic characteristics of participants in groups


**Variables**		**Non-PMS group (n = 237)**	**PMS group (n = 121)**	**P-value**
Age (yr)*		33.88 ± 7.04 (34, 10)	33.86 ± 8.06 (34, 13)	0.98
Work experience (yr)*		7.34 ± 6.24 (6, 10)	8.16 ± 6.85 (8, 11)	0.26
BMI (kg/m^2^)*		25.57 ± 4.62 (25.62, 5.47)	25.29 ± 5.54 (24.98, 6.14)	0.62
Children number*		1.19 ± 1.02 (1, 2)	1.11 ± 1.04 (1, 2)	0.43
Menarche (yr)*		13.64 ± 1.60 (14, 2)	13.46 ± 1.65 (14, 2)	0.33
Length of entire menstrual cycle (day) ${\;}^{*}$		25.60 ± 4.07 (25, 8)	25.38 ± 4.14 (25, 6)	0.64
Menstrual bleeding (days)*		6.13 ± 1.77 (7, 2)	6.38 ± 2.28 (7, 2)	0.26
Menstruation cycle**				
	**Regular**	181 (76.4)	94 (77.7)	
	**Irregular**	56 (23.6)	26 (21.5)	0.34
Occupation**				
	**Workers (n = 158)**	119 (75.3)	39 (24.7)	
	**Teachers (n = 200)**	118 (59.0)	82 (41.0)	0.002
Education**				
	**≤ Diploma**	116 (49.4)	42 (34.7)	
	**> Diploma**	119 (50.6)	79 (65.3)	0.01
Exercise**				
	**Yes**	29 (85.3)	5 (14.7)	
	**Irregular/No**	208 (64.2)	116 (35.8)	0.01
Marital status**				
	**Single**	37 (14.3)	23 (15.9)	
	**Married**	222 (85.7)	122 (84.1)	0.38
Oral contraceptive pill use**				
	**Yes**	14 (5.9)	4 (3.3.)	
	**No**	222 (94.1)	117 (96.7)	0.32
Antidepressant use**				
	**Yes**	7 (3.0)	8 (6.6)	
	**No**	230 (97.0)	113 (93.4)	0.16
*Data presented as Mean ± standard deviation (MD, IQR), Student's *t* test. **Data presented as n (%), Chi-square test/Fisher's Exact test. PMS: Premenstrual syndrome, BMI: Body mass index				

**Table 2:** Comparison of work productivity, ability to perform daily activities, and functional work capacity between groups


**Variables**	**Non-PMS group (n = 237)**	**PMS group (n = 121)**	**P-value**
**Work productivity**	1.2 ± 1.8 (0, 2)	3.4 ± 2.4 (3, 3)	< 0.001
**Ability to perform ADL**	2.0 ± 2.3 (1, 3)	4.2 ± 2.5 (5, 3)	< 0.001
**Functional work capacity**	6.4 ± 1.8 (7, 3)	3.8 ± 2.3 (4, 4)	< 0.001
Data presented as Mean ± SD (MD, IQR). Mann-Whitney test. PMS: Premenstrual syndrome, ADL: Activities of daily living			

**Table 3:** Multivariate linear regression coefficient of the PMS status and other factors according to functional work capacity, daily activities, and work productivity


	**Functional work capacity**		**Ability to perform daily activities**		**Work productivity**	
**Variables**	**B (95% CI)**	**P-value**	**B (95% CI)**	**P-value**	**B (95% CI)**	**P-value**
	**PMS status**	-2.54 (-2.99, -2.08)	< 0.001	2.54 (2.00, 3.08)	< 0.001	2.27 (1.82, 2.72)	< 0.001
**Occupation**	-1.21 (-1.79, -0.62)	< 0.001	1.54 (0.88, 2.30)	< 0.001	1.35 (0.79, 1.90)	< 0.001
**Education**	-0.76 (-1.33, -0.19)	0.009	0.99 (0.35, 1.64)	0.002	0.82 (0.28, 1.37)	0.003
**Exercise**	-1.33 (-2.16, -0.51)	0.002	1.38 (0.44, 1.64)	0.004	1.34 (0.55, 2.14)	0.001
Multivariate linear regression, enter method, adjusted for age, body mass index, work experience, marital status, child number, and menstrual characteristics, oral contraceptive pill, and antidepressant use. B (95% CI): Beta (95% of confidence interval), PMS: Premenstrual syndrome						

## 4. Discussion

Due to the relatively high prevalence of PMS disorders, this study investigated the impact of these disorders on women's work disruption. This study showed a significant association between premenstrual disorders and work productivity, functional work capacity, and ability to perform ADL.

In the present study, among all the participants, the prevalence of PMS disorders was 33.8%, while the prevalence of PMS and PMDD separately were 27.4% and 6.4%, respectively. On the other hand, the prevalence of PMS/PMDD in teachers (67.8%) was significantly higher than in workers (32.2%).

In a meta-analysis in 2017, the overall prevalence of PMS in Iran was estimated 70.8%, and 68.9% among university students (2). A study on healthcare workers, such as nurses, indicated that PMS is more prevalent due to hard working conditions, stressful work, and shift working (7). Other studies have reported the prevalence of PMS to be 52.0% in Saudi Arabian nurses and 38.1% in Turkish nurses (3, 8). In other studies the prevalence of PMS among emergency nurses and operating room technicians were 67.5% and 57.1%, respectively (9, 10). In a study on 125 office workers in UK, more than 90% reported some PMS symptoms and 40% suffered from moderate to severe of PMS symptoms (1). In a study on 117 female doctors, the prevalence of PMS was reported to be 50.7%. This relatively high prevalence could be because female doctors are facing severe stress from the workload, high social demands, and effort to balance between work and their family (6). These differences could be explained partly by cultural differences, residence (rural or urban), study designs, type of diagnostic tool and a negative view of menstruation, and consequently, the limitations imposed on women's response to menstruation between different societies and countries (1).

The current research demonstrates that PMS poses had a significant adverse effect on work productivity, particularly in relation to the severity of symptoms. Among the participants, those with PMDD experienced the most pronounced negative impact on work outcomes. In a study involving 699 women, aged between 18 and 45, it was observed that women with PMS encountered more instances of workday loss and had a greater number of workdays with reduced productivity of at least 50% compared to their normal levels (1). Additionally, a significant correlation was found between PMS and its psychological symptoms, which were reported to affect work productivity (7, 11). In a 8-yr cohort study in Korea, conducted on 121,024 working women, there was higher prevalence of PMS among workers compared to the general population. Moreover, since occupational stress, occupational exposures, and improper work conditions have been considered as occupational risk factors for PMS, manual workers presented highest cumulative incidence of PMS during follow-up periods (2007–2015) compared with office or other workers (agriculture, fishery, and forestry) (7).

In another study on 1867 working women in the US, 45.2% of women reported having absenteeism during the menstruation. The mean absenteeism was reported 5.8 days per year. Participants reported negative impact of their menstrual cycle: on energy levels (89.3%), mood (86.9%), concentration (77.2%), interest in own work (71.6%), efficiency (68.3%), and relationship with colleagues (39.0%) (12). A study on female doctors showed that the overall work productivity loss was 39.6% for those with PMS compared to 18.1% for those without PMS (6). On the other hand, in other studies, no significant association has been observed between PMS and work productivity. The different results may be due to various methods of measuring work performance and productivity or different women studied in the country, workplace, and job type (1).

Our study showed that increase in PMS severity negatively affects the ability to perform ADL, functional work capacity, and productivity. A survey of 264 females involved in academic teaching showed that the effect of premenstrual symptoms on work capacity, worsening by severity of symptoms at work, and job performance was significantly different between PMS and non-PMS women. Even 50% of the subjects found that their relationship with their colleagues was disturbed due to premenstrual symptoms (13). A study of 134 nurses in Turkey found that nurses with PMS had a significantly lower work-related quality of life (3). The severity of PMS psychological symptoms was reported to correlate significantly with occupational impairment. On the other hand, women with PMS realized that their poor physical work environment (such as lack of refreshing and rest facilities, improper work design, and work organization) worsened their symptoms (14). PMS makes women more sensitive to external stressors (15), so in addition to the usual life stress, work stress and poor workplace conditions can also affect the work capacity and performance.

In this survey, like another study (16), PMS was seen more frequently in working women with higher educational levels like teachers. In another study on emergency nurses, it was found that PMS has a significant and inverse association with the education level (9). Also, work productivity, ability to perform ADL, and functional work capacity showed a significantly worse score in the teachers than sorting workers; work stress, more complex tasks, and increased work responsibility in teachers can cause or exacerbate the PMS symptoms. The difficulty of taking leave can also affect the working class.

In a qualitative study, it was seen that working women with PMDD have higher absenteeism and lower productivity than women without PMDD or with mild PMS. Working life of PMDD in women not only affects the premenstrual period but also on the postmenstrual phase. It creates a feeling of guilt or overcompensating behaviors, such as working long hours or completing work at home, which disrupts the balance between work and life and repeating this situation every month will eventually lead to quitting or changing jobs. This issue shows the need to improve the awareness and support of employers, researchers, and policymakers in this field (17). A systematic review showed that exercise irrespective of its type, may efficiently alleviate both physical and psychological symptoms of PMS (18).

In an experimental study, the PMS severity was reduced using the progressive muscle relaxation technique, which was performed twice a week for a month (19).

The employer, line managers, and occupational health professionals must be aware and train personnel to improve their awareness of negative effects of PMS on working women for better communication and support of them. On the other hand, providing health and safety programs about the topic of PMS for working women, can improve their self-awareness and inform them about available treatments and coping strategies (14).

Our study had some limitations: first, a causal relationship could not be established due to the study design (cross-sectional). Second, PMS and PMDD was retrospectively identified, so a recall bias is probable. Third, the effect of PMS on work was assessed by a self-reported method. The present study was one of the few studies that have been conducted on working women other than healthcare workers. Evaluation of premenstrual disorder in different age, different work type, social and cultural groups using appropriate assessment tools are recommended. Longitudinal studies with objective methods and larger samples assessing PMS's impact on a wide range of work outcomes as well as work-related factors are recommended. Interventional studies should be planned to increase productivity, work capacity, and improve the quality of life and sleep quality, modify shiftwork, reduce job stress, and increase job satisfaction in working women.

This study showed a significant association between premenstrual symptoms and worse work productivity, functional work capacity, and ability to perform ADL. Teachers have a higher prevalence of PMS and worse work performance than sorting workers, which can be due to higher education level, work stress, more complex tasks, and increased work responsibility in teachers.

##  Data availability

Data supporting the findings of this study are available upon reasonable request from the corresponding author.

##  Author contributions 

Z. Loukzadeh, N. Eslamy, and M. Dehghan designed the study and conducted the research. Z. Loukzadeh, N. Eslamy, and AH Mehrparvar monitored, evaluated, and analyzed the result of the study. Further, Z. Loukzadeh, N. Eslamy, AH Mehrparvar, and M. Dehghan reviewed the article. All authors approved the final manuscript and take responsibility for the integrity of the data.

##  Acknowledgments

Financial support for the research was provided by the Research Vice-Chancellor of Shahid Sadoughi University of Medical Sciences, Yazd, Iran (Grant number: 6137).

##  Conflict of Interest

The authors declare that there is no conflict of interest.
